# Advances in Imaging Technology of Anterior Segment of the Eye

**DOI:** 10.1155/2021/9539765

**Published:** 2021-02-23

**Authors:** Sang Beom Han, Yu-Chi Liu, Karim Mohamed-Noriega, Jodhbir S. Mehta

**Affiliations:** ^1^Department of Ophthalmology, Kangwon National University School of Medicine, Kangwon National University Hospital, Chuncheon, Republic of Korea; ^2^Singapore National Eye Centre, Singapore, Singapore; ^3^Singapore Eye Research Institute, Singapore, Singapore; ^4^Department of Ophthalmology, Yong Loo Lin School of Medicine, National University of Singapore, Singapore, Singapore; ^5^Department of Ophthalmology, University Hospital, Faculty of Medicine, Autonomous University of Nuevo Leon, Monterrey, Mexico

## Abstract

Advances in imaging technology and computer science have allowed the development of newer assessment of the anterior segment, including Corvis ST, Brillouin microscopy, ultrahigh-resolution optical coherence tomography, and artificial intelligence. They enable accurate and precise assessment of structural and biomechanical alterations associated with anterior segment disorders. This review will focus on these 4 new techniques, and a brief overview of these modalities will be introduced. The authors will also discuss the recent advances in research regarding these techniques and potential application of these techniques in clinical practice. Many studies on these modalities have reported promising results, indicating the potential for more detailed comprehensive understanding of the anterior segment tissues.

## 1. Introduction

Direct visualization of ocular surface tissue using conventional techniques, e.g., slit-lamp biomicroscopy, still remains the primary examination tool for anterior segment diseases [1]. Development of devices for anterior segment imaging, such as anterior segment optical coherence tomography (OCT), corneal topography, specular microscopy, confocal microscopy, ultrasound biomicroscopy, and ocular response analyzer, has enabled detailed objective observations of anterior segment structures that can contribute to improved anatomical and visual outcome after cornea, cataract, and refractive surgeries [2].

Development of newer technologies, such as corneal visualization Scheimpflug Technology (Corvis ST), Brillouin microscopy, and ultrahigh-resolution OCT (UHR-OCT), is expected to allow even more detailed visualization of anterior segment structures, which would allow even more understanding of anterior segment pathology. Artificial intelligence may also be useful for providing optimal diagnostic and treatment protocols by integrating findings obtained using various imaging modalities.

In this review, we aim to provide an overview of these 4 newer techniques and discuss the research advances and potential clinical application of these modalities.

### 1.1. Corneal Biomechanical Assessment Using Ultrahigh-Speed Imaging and Special Analytical Methods

The Corvis ST (OCULUS Optikgeräte GmbH, Wetzlar, Germany) is the integration of two measurements modalities, i.e., a noncontact tonometer with a collimated high-intensity air pulse and an ultrafast Scheimpflug camera, that is used for the assessment of biomechanical properties of the cornea [3–9].

In the device, a fixed pressure air pulse causes corneal deformation, while passing through two applanation moments; the corneal movement during the deformation process is recorded using an ultrafast Scheimpflug camera at a speed of 4330 frames/s [10]. In each examination, a series of 140 images with width of 8.5 mm is obtained in 33 ms [3, 10]. As the Corvis ST is capable of analyzing the whole process of dynamic corneal deformation, it enables calculation of various dynamic corneal response (DCR) parameters [3, 11, 12]. Parameters including “deformation” are calculated without compensating for whole eye movement (WEM), while the parameters including “deflection” compensate for the WEM [13].

As the air pulse is triggered, the cornea deforms inward through the moment of first corneal applanation (A1), [3] at which the length of the applanated cornea (A1 length), the velocity of the corneal apex (A1 velocity), time from the measurement beginning (A1 time), and corneal deflection amplitude (A1 DeflAmp), defined as the displacement of the corneal apex without the WEM, are measured [3].

Just prior to A1, deformation amplitude ratio (DA ratio) at 1 or 2 mm, i.e., central deformation divided by an average of the deformation 1 or 2 mm from center with maximum value, is measured, and deflection amplitude ratio (DefA ratio) at 1 or 2 mm can be calculated after compensation for WEM [3].

Initially, the moment during the cornea highest concavity (HC), parameters including radius of corneal curvature (HC radius), time from beginning to the moment of HC (HC time), maximum deformation amplitude (DA), corneal deflection area (HCDeflArea), corneal deflection amplitude (HCDeflAmp), delta arc length of the outer corneal edge between initial state and HC (HCdArclength), and distance between two corneal peaks at HC (peak distance) are measured [3, 9, 13]. The radius of corneal curvature at HC (curvature radius HC) and the maximum value of the integrated inverse of the corneal radius HC (InvRadMax) are also determined [3, 9, 13].

At the moment of the second applanation (A2), the time for the A2 (A2 time), length of the flattened cornea (A2 length), and the velocity of the corneal apex (A2 velocity) are measured [3]. The value of corneal displacement before and after deformation (WEMax; maximum WEM) can also be determined [14, 15]. Corneal thickness over the entire cornea including central corneal thickness (CCT) and intraocular pressure (IOP) data, including uncorrected and biomechanically corrected IOP (bIOP), were also assessed [16]. As IOP values have strong association with the age, CCT, and DCR parameters, [17] bIOP was calculated based on an algorithm designed to compensate for the effects of these factors [18]. Stiffness parameters (SP) can be calculated by dividing the loading (air pressure—bIOP) on the cornea by the displacement of the corneal apex at A1 (SP-A1) and HC (SP-HC), respectively [3]. Several studies have demonstrated that Corvis ST had high repeatability and reproducibility for measurement of CCT, IOP, bIOP, and DCR parameters [10, 14, 15].

**Figure 1:**
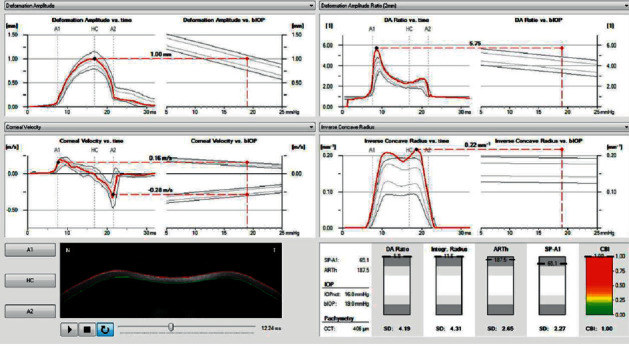
Vinciguerra screening report displays DCR parameters of a patient with keratoconus in comparison with normative values. The ARTh and SP-A1 are lower, and the DA ratio, integrated radius, and CBI are higher in keratoconus compared to normal subjects.

DCR parameters were shown to be helpful for the detection of corneal ectasia (Figure 1) [19, 20]. Keratoconus is associated with an increase in DA [21, 22]. A larger curvature radius HC and lower InvRadMax were related to increased corneal stiffness and higher resistance to deformation. [13].

For early and accurate diagnosis of corneal ectasia, several indices have been developed [6, 23]. Vinciguerra et al. [6] proposed the Corvis Biomechanical Index (CBI) by combining DCR parameters including the DA ratio at 2 mm, InvRadMax, and SP-A1 and corneal thickness data expressed as Ambrósio's Relational Thickness in the horizontal profile (ARTh) [23]. CBI with a cut-off value of 0.5 successfully detected 98.2% of corneal ectasia with 100% specificity, suggesting its potential value for early detection of keratectasia [6]. In a subsequent study, they presented 12 cases with subclinical keratoconus detected using CBI cut-off value of 0.5 in which topography and tomography were all normal [24].

**Figure 2:**
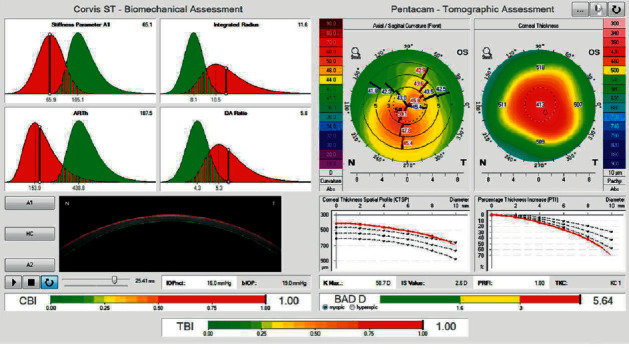
Corvis ST biomechanical/tomographic assessment of a keratoconic eye. Biomechanical assessment shows increase in DA ratio and integrated radius, and decrease in ARTh and SP-A1 (top left). Tomographic assessment shows central corneal thinning with an asymmetric bow tie pattern (top right). The percentage of thickness increase (PTI) graph shows an inferior escape from the normal mean. CBI, TBI, and BAD are all increased (bottom).

In 2017, Ambrosio et al. [23] introduced the tomographic biomechanical index (TBI) by integrating Scheimpflug-based corneal tomographic and biomechanical data to improve accuracy for detection of corneal ectasia (Figure 2) [23]. The TBI cut-off value of 0.79 provided 100% sensitivity and 100% specificity for detecting clinical corneal ectasia [23]. They also showed that TBI was significantly more accurate than CBI or Belin-Ambrosio Deviation display (BAD-D) for detecting corneal ectasia [23]. Steinberg et al. [25] also demonstrated that TBI was superior to CBI and BAD-D in keratoconus screening in topographical and tomographical normal fellow eyes of clinically ectatic eyes, although all the three indices were excellent for discriminating advanced keratoconus from normal eyes. Ferreira-Mendes et al. [26] revealed that the TBI was more accurate than BAD-D and CBI for detecting subclinical ectasia amongst topographically normal eyes in patients with asymmetric ectasia, indicating that the index might identify an intrinsic susceptibility for ectasia progression [26]. Other studies have also shown that TBI was the most accurate amongst the various indices developed so far for discriminating subclinical keratoconus from normal eyes [20,27]. Kataria et al. [20] reported that, among indices including CBI, TBI, BAD-D, and SP-A1, TBI showed the weakest correlation with biomechanical confounding factors. However, the cut-off value of TBI for detecting eyes with ectasia susceptibility varied amongst the studies, and no consensus regarding the cut-off value has been established yet [28]. Koh et al. [29] showed that 40% of cases with clinical ectasia in one eye and a fellow eye with normal topography were classified as normal by BAD-D, CBI, and TBI. These findings suggest that further studies are necessary for further development of indices and guidelines for discriminating eyes with ectasia susceptibility [29].

Corvis ST can also be helpful in monitoring changes in cornea after collagen cross-linking (CXL) treatment [30, 31]. CXL treatment was associated with increase in A2V and DA as well as decrease in A2L in eyes with keratoconus [31]. The difference between the A1L and A2L was reliable in discriminating cross-linked keratoconic corneas from untreated keratoconic or healthy corneas [31]. Hashemi et al. [30] showed that Corvis ST showed DCR changes suggesting corneal strengthening, such as decreased DA 2 mm and increased SP-A1, indicating that the device can provide biomechanical evidence of the efficacy of corneal CXL [30].

Corvis ST is also expected to be useful for evaluation of changes in corneal biomechanical properties associated with refractive errors and keratorefractive surgery [32–34]. Tubtimthong et al. [35] demonstrated that high myopia was associated with higher DA and smaller curvature radius, indicating that the condition might have reduced corneal stiffness and decreased stability. Hashemi et al. [34] showed that laser-assisted in situ keratomileusis (LASIK) led to more substantial changes in corneal biomechanical properties than photorefractive keratectomy (PRK) in high myopia, although both procedures caused significant biomechanical changes in the cornea. Corvis ST has shown that both LASIK and small incision lenticule extraction (SMILE) cause remarkable changes in corneal biomechanical parameters [32, 33, 36]. However, SMILE was associated with reduced change in DA and better recovery of corneal biomechanical strength [32, 33]. Khamar et al. [36] reported that creation of a LASIK flap caused greater acute biomechanical weakening intraoperatively in comparison to a SMILE cap, although both resulted in similar biomechanical changes after wound healing.

Cataract surgery was associated with decreased SP-A1 and increased DA even at 3 months postoperatively, suggesting decreased corneal stiffness [37, 38]. As the reduction in corneal stiffness was associated with falsely low IOP measurements, care should be taken particularly when evaluating glaucoma patients after cataract surgery [37].

Corvis ST is also expected to be a potential biomarker in thyroid orbitopathy [39, 40]. Thyroid orbitopathy was associated with a decrease in WEM, which had a correlation with increase in cross-sectional area of the extraocular muscles [40]. Leszczynska et al. [39] also demonstrated biomechanical alterations including decreased WEM length and time, increased bIOP, and higher SP, indicating reduced orbital compliance in association with thyroid orbitopathy [39].

With the development of OCT technology, swept source (SS) OCT combined with air puff applanation is also expected to enable accurate and precise evaluation of corneal biomechanical properties [41, 42]. Several studies have shown the efficacy of SS-OCT with an air puff in assessment of dynamic response of cornea to air pulse, suggesting it as a potential option for the in vivo assessment of corneal mechanical properties, particularly due to its high resolution [43, 44].

## 2. Brillouin Microscopy

Brillouin microscopy is a recently introduced modality to measure the viscoelastic property of the cornea in vivo [45]. In Brillouin microscopy, a low-power, near-infrared laser beam is focused into the corneal tissue and it interacts with intrinsic acoustic waves, which leads to a Brillouin frequency shift of scattered light reflected from the modulation of the focus [45, 46]. The Brillouin frequency shift is proportional to the acoustic propagation speed of tissue at the focus, which is proportional to the square of the longitudinal modulus; thus, assessment of the Brillouin frequency shift provides a determination of longitudinal modulus or mechanical compressibility, which is the inverse of the longitudinal modulus, of the target tissue [45].

Brillouin microscopy is advantageous due to its noncontact nature and ability to generate 3D mapping of the spatial variation of longitudinal modulus using high-resolution confocal spectrometer and is expected to be widely used for practice and research on anterior segment disorders [45, 46].

Clinical studies using Brillouin microscopy have demonstrated significant alteration in corneal elastic properties in keratoconus, suggesting the potential applicability of the device for detection of cornea with ectasia susceptibility [47, 48]. Brillouin frequency shift in the cone region is significantly reduced in keratoconic corneas compared to normal ones [47–49]. In keratoconus, the cone region has substantially decreased Brillouin frequency shift, compared to the peripheral regions [47–49]. Shao et al. [47] also demonstrated that asymmetry of Brillouin frequency shifts between the right and left cone regions is significantly higher in eyes with early keratoconus compared with normal eyes, indicating that bilateral symmetry of Brillouin value might have a value for detection of early-stage KC.

The modality may also be useful in evaluation of corneal CXL protocols [50, 51]. Brillouin analyses revealed that accelerated CXL protocols were especially ineffective in the deeper portions of the cornea [50], and the stiffening effect of localized-CXL extended to regions surrounding the localized irradiated area [51].

Brillouin microscopy can be useful in the management of corneal endothelial disorders and monitoring the disease severity [52, 53]. Brillouin frequency shift was shown to have negative correlation with corneal hydration, [52] which might be helpful in evaluating abnormal hydration change associated with endothelial dysfunction. Eltony et al. [53] revealed that patients with Fuchs' endothelial dystrophy showed a centralized reduction in Brillouin shift, which was consistent with central corneal edema. Brillouin microscopy also detected substantially reduced corneal hydration after Descemet membrane endothelial keratoplasty (DMEK) [53].

The technique is also expected to be useful for the evaluation of corneal biomechanical change associated with cornea, refractive, and cataract surgery [45, 54]. LASIK flap creation resulted in significantly reduced Brillouin frequency shift, due to reduced corneal stiffness [54]. As differences in biomechanical properties including corneal hydration might contribute to the variability in refractive outcome after cataract and refractive surgeries, [55, 56] Brillouin microscopy is expected to be helpful for establishment of individually tailored nomograms for optimal visual outcome [45, 55].

## 3. Ultrahigh Resolution OCT (UHR-OCT)

Although anterior segment time-domain OCT, which has been commercially utilized since the early 2000s, is capable of providing comprehensive images of anterior segment structure, it lacks the ability to show structural details due to the lower resolution [57, 58]. Advances in technology have enabled development of spectral-domain OCT with improved axial resolution of 4–7 *μ*m and, subsequently, ultrahigh-resolution (UHR) OCT with axial resolution of 1–4 *μ*m [58].

UHR-OCT uses a light source based on Ti:sapphire laser with a broad bandwidth of larger than 100 nm as well as an optical system specifically designed to deliver optimal performance [59], which results in a resolution of less than 5 *μ*m [58, 59].

Enhanced axial resolution of UHR-OCT enabled precise delineation of all 5 corneal layers and thickness measurement of each layer [59]. The device also allowed visualization of microstructures, such as limbal palisades of Vogt, limbal blood vessels, corneal nerve fiber bundles, and aqueous humor drainage pathway including intrascleral, episcleral, and conjunctival venous plexuses [59], which may be helpful for understanding the pathophysiology of various anterior segment disorders and glaucoma.

UHR-OCT also allows visualization of precorneal tear film and tear film lipid layer [60–62] and provides thickness data of tear film and lipid layer with excellent reproducibility [61, 62]. These findings suggest that the UHR-OCT can be a viable option for diagnosis and management of dry eye disease [59]. It also enabled precise evaluation of re-epithelialization after corneal injury by 3D mapping and observation of microarchitectural alterations in early phases of corneal wound healing [63, 64].

UHR-OCT was also shown to be a viable tool for detection, differential diagnosis, and monitoring of treatment response of ocular surface tumors including ocular surface squamous neoplasia and melanoma [58, 65, 66]. It can provide clear demarcation and information regarding depth, localization, and characteristics of various ocular surface lesions [67]. UHR-OCT findings of the lesions showed close correlation with histopathologic features [66, 67]. Shousha et al. [66] suggested that UHR-OCT can play a critical role in guiding the diagnosis in some cases, in which the optical signs obtained using the device indicated that the presumed clinical diagnosis might be incorrect and favored a diagnosis later confirmed by histopathologic examination [66]. These findings indicate that UHR-OCT may have potential for noninvasive options for confirming diagnosis and monitoring treatment response of ocular surface lesions [66, 67]. The modality also enables detection of subclinical ocular surface neoplasia that cannot be observed by slit-lamp examination [66, 68], which may be invaluable for surveillance for recurrent or residual tumors after treatment [58].

UHR-OCT can also be helpful for diagnosis and treatment of ocular surface infection. For instance, the device allows visualization of characteristic signs of *Acanthamoeba* keratitis, such as corneal nerve thickening reflecting radial keratoneuritis and highly reflective dots indicating the cysts [59].

The ability of the UHR-OCT to generate vertical thickness map and indices of the corneal epithelium and Bowman's layer can be helpful for discrimination of subclinical corneal ectasia [69, 70]. In 2019, Santos et al. [71] reported that a UHR-OCT combined with a deep learning algorithm called CorneaNet was capable of segmentation of both healthy and keratoconus images with high reliability, suggesting that the device can be a useful tool for early detection of keratoconus.

UHR-OCT allows in vivo high-resolution visualization of corneal endothelial cells and measurement of density of the cells [72], which can be beneficial for detection and monitoring of pathologic conditions in endothelium, e.g., endothelial guttata in Fuchs' endothelial dystrophy [68].

The device can also be helpful for management after corneal surgery, particularly after keratoplasty. UHR-OCT allows visualization of endothelial graft after DMEK [59], which enables early detection of graft detachment. It can also detect a gap in the keratoprosthesis-cornea interface with absence of epithelial closure after implantation of artificial cornea [73], which is critical for prevention of complications, such as leakage, graft extrusion, and endophthalmitis [73].

As UHR-OCT technology has a potential for visualization of anterior segment structure at a microscopic level and assessment of ocular biometry with excellent accuracy, it is expected to further improve visual outcome after cataract and refractive surgery [2].

## 4. Artificial Intelligence

Artificial intelligence (AI) using machine learning and deep learning is expected to be helpful for diagnosis and treatment of anterior segment diseases, although the use of AI has already been extensively established for systemic associations with retinal findings [74, 75]. Machine learning algorithms including support vector machines or random forest models are programmed to adapt according to the input data and produce assumptions, e.g., determinations or predictions, based on the parameters of its algorithm [76]. Conventional machine learning might be sufficient for designing predictive algorithms using clinical data including medical records or population-based studies [77]. Deep learning refers to a subset of machine learning technique that involves neural networks comprising multiple neuron-like computational layers of algorithms, i.e., convolutional neural networks (CNNs) [76]. Deep learning has been widely used for the analysis of image-based data including anterior segment photographs, fundus photographs, or OCT images, due to its improved diagnostic performance [77].

Mahesh Kumar et al. [78] reported that a multiclass computer-aided system, based on machine learning using support vector machine by sequential minimal optimization algorithm, showed accuracy of 97% for diagnosing anterior segment eye abnormalities such as senile arcus or cataracts, suggesting the potential of ophthalmic image analysis using AI for clinical application.

AI has currently been useful for development of indices for discrimination of keratoconus [23,79]. The TBI developed using random forest model with leave-one-out cross-validation was shown to be superior to other indices, such as CBI and BAD-D, for differentiation between keratoconus and normal corneas [23, 26]. The Pentacam random forest index (PRFI), a random forest model generated using Pentacam HR (Oculus, Wetzlar, Germany) data, was also demonstrated to improve the accuracy of detection of ectasia susceptibility compared to BAD-D [79].

AI also allows rapid assessment of the corneal endothelium with good reliability [80–83]. A deep learning method called *U*-net was capable of substantially faster and more accurate segmentation compared to manual segmentation [80, 81]. Heinzelmann et al. [84] revealed that the endothelial cell counts measured using U-Net showed strong correlation with those obtained with the gold standard, suggesting the potential applicability of the AI model in the long-term assessment of corneal grafts. After DMEK, deep learning model using CNN can also be useful for automated quantification of graft dislocation, which may enable early detection of graft [85].

Al enables rapid and accurate evaluation of corneal subbasal nerve plexus using in vivo confocal microscopy (IVCM) [86, 87]. Using neural network and random forest models, Chen et al. [86] generated an automated method for detection and quantification of nerve fibers in IVCM mages with speed and repeatability superior to manual quantification. Al-Fahdawi et al. [88] introduced an automatic system using AI for nerve segmentation and assessment of parameters including nerve thickness, tortuosity, and length in IVCM images, which is expected to be useful for early detection of diabetic peripheral neuropathy. Williams et al. [87] also introduced a deep learning algorithm for the automated quantification of the corneal nerves, which showed rapid and excellent localization performance.

AI can be helpful for the diagnosis and management of ocular surface infection [89,90]. Xu et al. [89] revealed that an automatic hyphae detection method based on image recognition with adaptive robust binary pattern in IVCM images was more accurate than corneal smear examination, suggesting the potential applicability of AI for noninvasive diagnosis of fungal keratitis [89]. A system for automatic segmentation of corneal ulcer areas using a joint method of Otsu and Gaussian mixture modeling has also been proposed [90].

In dry eye disease, deep learning can be applied for the automatic segmentation of the anterior segment OCT image with a thresholding-based segmentation algorithm for the evaluation of the tear meniscus [91].

For iris tumor, Ouabida et al. [92] showed that an automatic method using the Vander Lugt correlator based active contour method and a *K*-means clustering model detected all iris tumors with an accuracy of 100% [92].

AI is also expected to be useful for screening of cataract [93–95]. Lin et al. [93] introduced an automatic detection protocol for pediatric cataracts using a deep learning algorithm using anterior segment photographs. Yang et al. [94] also developed an ensemble learning based method using support vector machine and backpropagation neural network, which showed good performance for detection and grading of cataract [94]. Wu et al. [95] reported that a universal AI platform integrated with a AI-based multilevel collaborative pattern showed excellent reliability for diagnosis of cataract and detection of referable cases, which might enable effective referral service for cataracts. Machine learning algorithms have also shown higher efficacy with comparable safety in nomogram prediction in SMILE compared with surgeon‐developed normograms [96].

## 5. Conclusion

Novel techniques including Corvis ST, Brillouin microscopy, and UHR-OCT are expected to enable even more detailed assessment of anterior segment structures with high accuracy. AI can integrate the findings from these new modalities as well as from conventional imaging devices and generate protocols for optimal diagnosis and treatment of various anterior segment disorders.

With further developments, these future techniques may allow comprehensive and precise evaluation of anatomical and functional alterations associated with various anterior segment diseases, which would be critical for enhanced diagnostic performance and treatment outcome.

## Data Availability

The data supporting this systemic review are from previously reported studies and datasets, which have been cited in this article.
